# Unveiling the Hidden Burden: Estimating All-Cause Mortality Risk in Older Individuals with Type 2 Diabetes

**DOI:** 10.1155/2024/1741878

**Published:** 2024-01-20

**Authors:** Dikang Pan, Hui Wang, Sensen Wu, Jingyu Wang, Yachan Ning, Jianming Guo, Cong Wang, Yongquan Gu

**Affiliations:** ^1^Xuanwu Hospital, Capital Medical University, Beijing, China; ^2^Renal Division, Peking University First Hospital, Beijing, China

## Abstract

**Background:**

The mortality rate among older persons with diabetes has been steadily increasing, resulting in significant health and economic burdens on both society and individuals. The objective of this study is to develop and validate a predictive nomogram for estimating the 5-year all-cause mortality risk in older persons with T2D (T2D).

**Methods:**

We obtained data from the National Health and Nutrition Survey (NHANES). A random 7 : 3 split was made between the training and validation sets. By linking the national mortality index up until December 31, 2019, we ensured a minimum of 5 years of follow-up to assess all-cause mortality. A nomogram was developed in the training cohort using a logistic regression model as well as a least absolute shrinkage and selection operator (LASSO) regression model for predicting the 5-year risk of all-cause mortality. Finally, the prediction performance of the nomogram is evaluated using several validation methods.

**Results:**

We constructed a comprehensive prediction model based on the results of multivariate analysis and LASSO binomial regression. These models were then validated using data from the validation cohort. The final model includes four independent predictors: age, gender, estimated glomerular filtration rate, and white blood cell count. The C-index values for the training and validation cohorts were 0.748 and 0.762, respectively. The calibration curve demonstrates satisfactory consistency between the two cohorts.

**Conclusions:**

The newly developed nomogram proves to be a valuable tool in accurately predicting the 5-year all-cause mortality risk among older persons with diabetes, providing crucial information for tailored interventions.

## 1. Introduction

The aging of the world's population is rapidly accelerating, with the number of people over 65 years old increasing from 461 million in 2004 to an estimated 2 billion in 2050. This demographic shift has a profound impact on the planning and provision of health and social care [1, 2]. Aging is the primary risk factor for chronic diseases such as cancer, cardiovascular diseases, diabetes, and neurodegenerative diseases. These diseases now disproportionately affect the elderly population and can impair sensory, motor, and cognitive functions, leading to a reduced quality of life [3]. The biggest medical challenge in treating a growing number of elderly patients is dealing with multiple diseases [4, 5]. At least half of elderly individuals over 70 years old suffer from multiple diseases, leading to the concurrent use of five or more medications (known as multidrug syndrome). This phenomenon accounts for over 10% of the general population and 30% of the elderly population [6, 7]. Furthermore, as the incidence rate of chronic diseases continues to rise, the demand for health and social care services will also increase, resulting in an impact on health expenditure [8].

Currently, there are approximately 537 million adults worldwide who suffer from diabetes, with over 90% of them being T2D (T2D) patients. This has resulted in a high prevalence rate of adult diabetes, reaching 10.5% [9]. The mortality risk for individuals with diabetes is 2-4 times higher compared to nondiabetic individuals [10, 11]. Epidemiological studies have shown that diabetes-related mortality is continuing to rise, particularly among the elderly population. The far-reaching impact of diabetes on mortality places a heavy burden on families and society [12]. Early identification of high-risk groups and implementation of intervention measures can help reduce the risk of premature death among elderly patients with diabetes. Therefore, it is crucial to establish a mortality prediction model specifically for elderly patients with diabetes. Although a few studies have developed mortality prediction models for the diabetic population [13], these studies were limited in terms of their research population, follow-up duration, and models used to calculate mortality risk. Consequently, these models were not applicable to the general elderly population with diabetes. To date, there have been no population-based studies aimed at developing a risk prediction model for mortality in elderly individuals with diabetes.

A nomogram is a visual statistical prognostic tool that is widely used in the clinical evaluation of prognosis by calculating scores based on potential predictive factors [14]. It can provide a quick assessment of clinical risk stratification and prognosis judgment [15]. The objective of this study is to establish and validate a 5-year all-cause mortality prediction nomogram for elderly diabetes patients based on the American population.

## 2. Methods

### 2.1. Study Design and Population

The National Health and Nutrition Examination Survey (NHANES) is an ongoing research project that provides estimates of the population's nutrition and health status in the United States. This survey uses a stratified, multistage probability design to recruit a representative sample of the American population. Data is gathered through structured interviews with individuals at home, health screenings at mobile health screening centers, and laboratory sample analysis [16].

**Figure 1:**
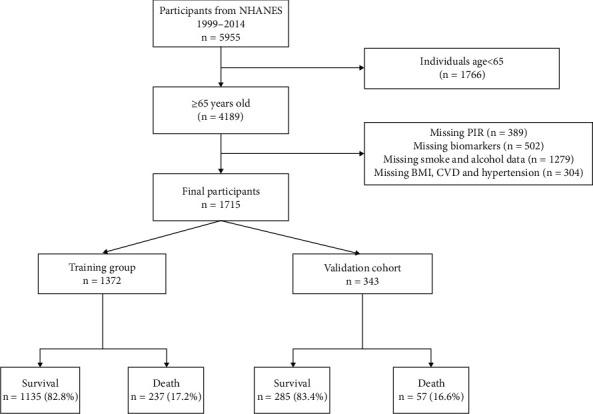
Flow chart of the training and validation cohorts. PIR: poverty-to-income ratio; BMI: body mass index; CVD: cardiovascular disease.

Participants were diabetes patients aged 65 years and above. T2D was defined as a diagnosed case of diabetes mellitus with insulin or oral hypoglycemic agents and fasting glucose levels above 7.0 mmol/L (126 mg/dL) or glycated hemoglobin A1c (HbA1c) levels above 6.5% [17]. Participants without follow-up results and information on key candidate variables were excluded. The detailed selection process is shown in Figure 1. The follow-up all-cause mortality was determined using the national death index up until December 31, 2019. The training and validation cohorts were selected to provide at least 5 years of follow-up for assessing all-cause mortality.

### 2.2. Potential Predictors

Information on participants' sociodemographic characteristics, smoking status, alcohol consumption, use of diabetes medication, and hypertension was collected using a standardized questionnaire. Participants who had smoked fewer than 100 cigarettes during their lifetime were classified as nonsmokers, while those who had smoked more than 100 cigarettes in the past but had not quit were defined as current smokers. Former smokers were those who had smoked more than 100 cigarettes in the past but had quit. Drinking status was categorized into three levels: nondrinker, low to moderate drinker (less than 2 drinks per day for men and less than 1 drink per day for women), and heavy drinker (2 or more drinks per day for men and 1 or more drink per day for women). Race/ethnicity was classified as non-Hispanic white or other. Educational attainment was categorized as less than high school, high school or equivalent, or college or higher. Poverty income ratio (PIR) scores were defined as 0-1.0, 1.0-3.0, and greater than or equal to 3.01. BMI was calculated as weight divided by height squared (kg/m^2^) and is classified as <25.00, 25.0-29.99, and greater than or equal to 30.00. We also included a number of laboratory markers including lymphocytes, total cholesterol, triglycerides, uric acid, estimated glomerular filtration rate measured by creatinine, albumin, alanine aminotransferase, and aspartate aminotransferase. All of the above indicators were obtained from the NHANES database and measured as previously described in the literature [15, 18]. To avoid possible bias, variables were excluded if they had more than 20% missing values. Variables with less than 20% missing data were processed for multiple imputations using the random forest algorithm (trained by other nonmissing variables) through the “mice” package of RStudio software [19, 20].

### 2.3. Statistical Analyses

Statistical analyses were performed using SPSS (Version 26; IBM Corp, Armonk, NY) and RStudio software. *P* value < 0.05 (two-sided) were considered significant. Patients were randomly divided at a ratio of 4 : 1 into the training and validation cohort. Differences between them were analyzed. Categorical variables were presented as numbers and percentages, and continuous variables were presented as mean ± standard deviation (SD). Differences between the two cohorts were explored using the chi-squared test for categorical variables and the independent *t*-test for continuous variables.

To construct nomograms, we compared differences between whether patients with T2D died at 5 years in the training cohort and then used multivariate logistic regression analyses to identify independent factors for T2D, including variables with a *P* value of < 0.05 in univariate analyses; the odds ratio (OR) and 95% confidence interval (CI) of each risk factor in the logistic regression model were calculated. Finally, in a linear regression model, the least absolute shrinkage and selection operator (LASSO) regression analysis method is utilized for shrinkage and variable selection. Firstly, the data is analyzed using the training set and the LASSO regression method. The LASSO regression analysis is then applied to select four independent variables based on lambda.min, which determines effective risk predictors suitable for predicting 5-year all-cause mortality in individuals with T2D.

Performances of the nomogram model were assessed in the training and validation cohorts, respectively. Firstly, the performance of the nomogram model was evaluated in the training and validation cohorts, respectively. First, the discriminative power of the nomogram was evaluated using the area under the curve (AUC) of the receiver operating characteristic curve (ROC). An AUC of 1.0 was considered to indicate that the nomogram had perfect discrimination ability. Secondly, the calibration of the nomogram was evaluated by the Hosmer-Lemeshow goodness-of-fit test (*P* > 0.05 indicates good calibration) [21]. Thirdly, by plotting the calibration curve, we analyzed the relationship between observed and predicted probability in the training and validation cohort. Moreover, a model for predicting the maximum net benefit [22] was developed using the decision curve analysis (DCA) method.

## 3. Results

### 3.1. Baseline Characteristics and Predictors of Mortality

The final study included 1372 participants in the training cohort and 343 participants in the validation cohort. Over a 5-year follow-up period, 237 cases (17.3%) in the training cohort and 57 cases (16.6%) in the validation cohort resulted in death. The descriptive statistics for both groups are presented in Table 1.

**Figure 2:**
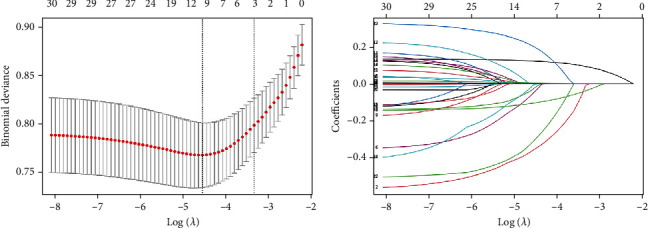
(a) Cross-validation plot for the penalty term. (b) Plot for LASSO regression coefficients.

In the univariate logistic regression model, all potential predictors, except for race, education level, smoking status, alcohol consumption status, PIR, hypertension, CVD, lymphocytes, total cholesterol, uric acid, total cholesterol, ALT, and HDL, showed an association with mortality (Table 2). Table 2 displays the models constructed using univariate and multivariate logistic regression for all candidate predictive factors. Additionally, the relevant characteristic variables mentioned above were included in the LASSO regression analysis (Figures 2(a) and 2(b)). Based on the data from the development group, four nonzero potential predictive factors were selected from the results of the LASSO regression analysis. These factors were age, gender, albumin, and EGFR. Ultimately, the predictive model was constructed using the combined results of multivariate logistic regression and LASSO regression.

### 3.2. Development of Nomogram

**Figure 3:**
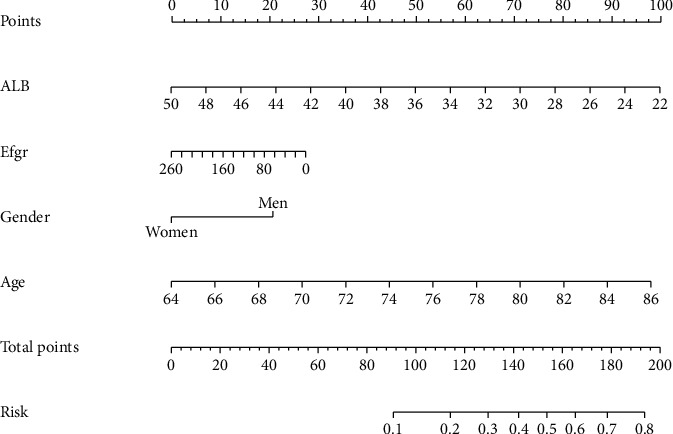
Nomogram of 5-year all-cause mortality in elderly patients with diabetes mellitus.

According to the results of the final model, we have constructed a nomogram for predicting the probability of all-cause mortality in elderly individuals with diabetes over a 5-year period (Figure 3). The column chart consists of seven axes, where axes 2-5 represent each prognostic factor included in the final model. Each predictor is allocated a different weighted score in the nomogram. Axes 6-7 demonstrate that a higher total score is indicative of an increased risk of all-cause mortality over the course of five years.

### 3.3. Internal and External Validation

**Figure 4:**
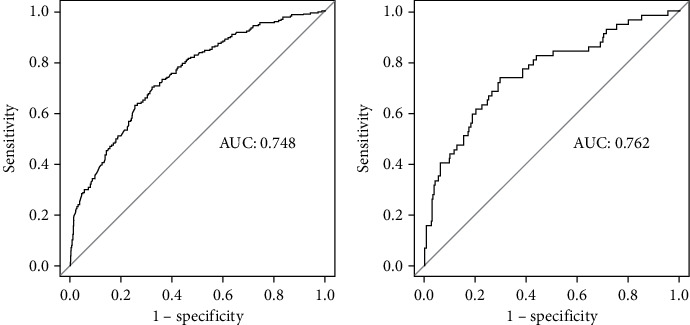
(a) ROC curve for the training cohort. (b) ROC curve for the validation cohort.

**Figure 5:**
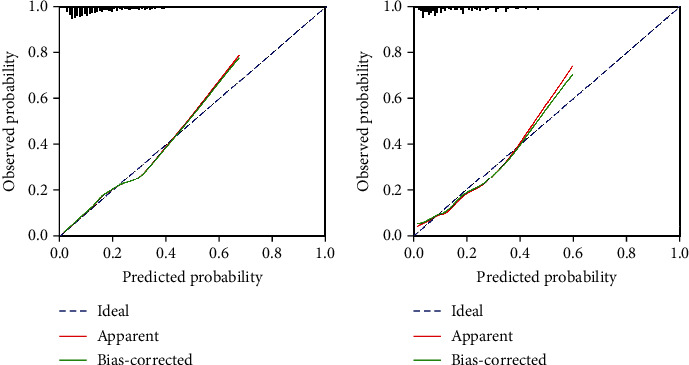
(a) Calibration curve for the training cohort. (b) Calibration curve for the validation cohort.

We used the receiver operating characteristic (ROC) curve to assess the discriminability of the model. In the training cohort, the AUC of the model was 0.748 (95% CI: 0.705-0.791) (Figure 4(a)). The calibration curve, which closely follows the diagonal, indicates good consistency between the predicted results of the model and the actual results (Figure 5(a)). In the validation cohort, the AUC of the model was 0.762 (95% CI: 0.694-0.831) (Figure 4(b)). Additionally, the calibration chart demonstrates that the model fits well with the 5-year all-cause mortality rate (Figure 5(b)).

**Figure 6:**
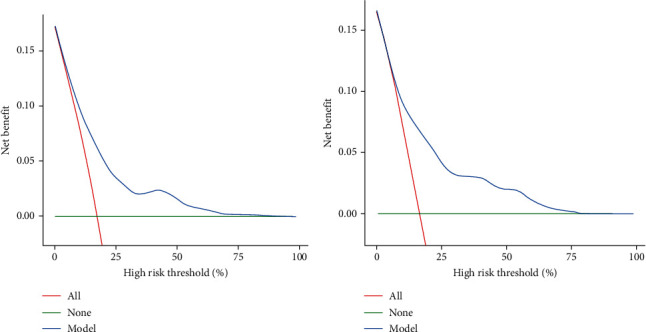
(a) DCA curve for the training cohort. (b) DCA curve for the validation cohort.

Figure 6 presents the results of the decision curve analysis (DCA) curve for both the development and validation groups. The dashed line represents the model, the gray line represents the net benefit for all patients with DR, and the black line represents the net benefit for patients without DR. The area between the black and gray lines in the model curve represents the clinical applicability of the model. If the dashed line is above the black and gray lines, it indicates that the range of values covered by the dashed line provides benefits.

## 4. Discussion

In the NHANES follow-up cohort, our study developed and validated a novel and practical nomogram diabetes prediction model for estimating the 5-year risk of all-cause death from T2D in older adults. We used the logistic regression model and lasso regression to identify four factors predicting 5-year mortality: age, sex, EGFR, and albumin. The model revealed that male sex, older age, higher EGFR, and lower albumin were key factors in determining the 5-year all-cause mortality of T2D patients, which were consistent with risk factors reported in previous studies [23–26].

In recent years, nomograms have been increasingly utilized to diagnose and predict various diseases, including cancer [27], myocardial infarction [28], and hypertension [15]. Utilizing nomograms simplifies the interpretation of relevant risk factors, aiding clinicians and patients in navigating disease challenges. With the increasing life expectancy and the growing population of elderly patients with diabetes, it is imperative to develop a universal risk assessment tool for all-cause mortality in this population. However, no previous study has constructed a nomogram to predict 5-year all-cause mortality in elderly patients with diabetes. Therefore, our study is aimed at constructing a prognostic nomogram incorporating demographic characteristics and routine laboratory parameters, providing important prognostic information to guide the development of individualized intervention strategies aimed at reducing the risk of premature death in older patients with diabetes.

One important result of this study is the internal and external validation of our model. We observed that the nomogram exhibited a discrimination ability greater than 0.7 in distinguishing 5-year all-cause death from T2D, and the predicted probability of all-cause death closely aligned with the actual probability along the 45-degree diagonal. These findings demonstrate the effectiveness of our prediction model.

According to our study, the 5-year all-cause mortality of elderly patients with T2D was negatively correlated with albumin levels, suggesting that worse body nutrition is associated with higher mortality rates. Many studies have confirmed the impact of high and low albumin levels on the survival of diabetic patients, with the mortality rate being higher in the group with low albumin levels [29, 30]. Arques [31] reported a reduced risk of T2D with high serum albumin concentrations. Similar to previous studies, our study found a negative correlation between serum albumin concentration and T2D, as well as associations between serum albumin concentration and the prognosis of cardiovascular disease [31], cancer mortality [32], and all-cause mortality [33]. Additionally, in our study, T2D participants with higher uric acid (UA) levels had higher all-cause mortality rates.

Although many previous studies have addressed the relationship between estimated glomerular filtration rate (EGFR) and all-cause mortality, most of them have focused on the general population [34, 35]. Some studies, however, have investigated the relationship between EGFR and all-cause mortality specifically in diabetic patients. For instance, in a large prospective study in China that recruited 4421 patients, all-cause mortality increased from 1.2% (95% CI 0.8-1.7) to 18.3% (9.1-27.5) (*P* < 0.001) after a median follow-up period of 39.4 months, as renal function deteriorated from stage 1 (EGFR ≥ 90 mL/min/1.73 m^2^) to stage 4 (15-29 mL/min/1.73 m^2^) [36]. Similarly, the results of another randomized controlled trial involving 8879 patients showed that in diabetic patients, an annual sharp decline in EGFR was significantly associated with the risk of all-cause mortality [37]. These findings align with our results regarding the relationship between EGFR and all-cause mortality in diabetic patients based on the prediction model.

Age emerged as the most influential risk factor for diabetes-related death. In a large cohort study involving 435,369 diabetic patients, it was found that all-cause mortality and cardiovascular mortality increased exponentially with age, consistent with a Swedish study in 2015 [38]. A recent study in Australia included 743,709 diabetic patients registered from 1997 to 2011 and explored the impact of age at diagnosis and disease duration on diabetes mortality. The study ultimately found that young-onset T2D increased the risk of death, primarily through early cardiovascular disease death. Therefore, efforts to delay the onset of T2D may help reduce mortality rates [39].

Gender is also an important factor in T2D-related death. Wang et al. studied 2535 NHANES participants with confirmed diabetes between 1999 and 2018 and observed that the risk of all-cause mortality and cardiac mortality was significantly higher in men compared to women, with or without diabetes. Male patients with T2D also have a higher risk of microvascular and macrovascular complications compared to female patients with diabetes. Previous studies have suggested that sex hormones, like estrogen and androgen, may contribute to the sex difference in diabetes-related mortality. The relative risk for women may also be higher, especially for mortality related to cardiovascular disease and kidney disease [40, 41].

However, our study has certain limitations. Firstly, it is important to note that except for selected variables in the questionnaire survey, all of our data originates from the health examination conducted by NHANES in the family interview and mobile screening center. This reliance on a single data source may introduce potential inaccuracies and compromise the objectivity of our results. Secondly, certain potential predictors, such as diet and exercise, were not considered in our model. This omission limits the comprehensiveness of our analysis and may impact the overall findings. Thirdly, due to the extensive database of NHANES variables, it was not feasible to include all relevant covariates related to diabetes. Consequently, some important variables might have been overlooked during the selection process. Fourthly, the lack of follow-up of some diabetic patients in our study may have affected the results of the nomogram. Lastly, our study lacks external validation. In order to establish the reliability of our findings, it is necessary to validate the results using external datasets.

## 5. Conclusion

The newly developed nomogram proves to be a valuable tool in accurately predicting the 5-year all-cause mortality risk among elderly patients with diabetes, providing crucial information for tailored interventions.

## Figures and Tables

**Table 1:** Baseline characteristics in training and validation cohorts.

Variables	Total	Training cohort	Validation cohort	*P*
	*N* = 1715	*N* = 1372	*N* = 343	
Age (years)	73.11 ± 5.56	73.16 ± 5.62	72.93 ± 5.30	0.649
Sex, *n* (%)				0.405
Male	857 (49.97)	693 (50.51)	164 (47.81)	
Female	858 (50.03)	679 (49.49)	179 (52.19)	
Race, *n* (%)				0.810
Non-Hispanic White	710 (41.4)	573 (41.76)	137 (39.94)	
Non-Hispanic Black	368 (21.46)	296 (21.57)	72 (20.99)	
Other Hispanic	440 (25.66)	345 (25.15)	95 (27.70)	
Other races	197 (11.49)	158 (11.52)	39 (11.37)	
Education level, *n* (%)				0.633
Less than high school	700 (40.82)	567 (41.33)	133 (38.78)	
High school diploma or GED	409 (23.85)	327 (23.83)	82 (23.91)	
More than high school	606 (35.34)	478 (34.84)	128 (37.32)	
Smoking status, *n* (%)				0.694
Never smoker	849 (49.5)	677 (49.34)	172 (50.15)	
Ever smoker	545 (31.78)	442 (32.22)	103 (30.03)	
Current smoker	321 (18.72)	253 (18.44)	68 (19.83)	
Drinking status, *n* (%)				0.298
Nondrinker	886 (51.66)	699 (50.95)	187 (54.52)	
Low-to-moderate drinker	174 (10.15)	146 (10.64)	28 (8.16)	
Heavy drinker	655 (38.19)	527 (38.41)	128 (37.32)	
BMI (kg/m^2^), *n* (%)				0.965
<25.00	343 (20)	276 (20.12)	67 (19.53)	
25.00–29.99	637 (37.14)	508 (37.03)	129 (37.61)	
≥30.0	735 (42.86)	588 (42.86)	147 (42.86)	
PIR, *n* (%)				0.470
<1.0	314 (18.31)	250 (18.22)	64 (18.66)	
1.0-3.0	927 (54.05)	751 (54.74)	176 (51.31)	
>3.0	474 (27.64)	371 (27.04)	103 (30.03)	
Medication use				0.629
No	586 (34.17)	465 (33.89)	121 (35.28)	
Yes	1129 (65.83)	907 (66.11)	222 (64.72)	
Hypertension	1190 (69.39)	935 (68.15)	255 (74.34)	0.062
CVD	187 (10.9)	143 (10.4)	44 (12.8)	0.469
Antihyperlipidemic drug	730 (42.57)	572 (41.69)	158 (46.06)	0.076
Lymphocyte	2.03 ± 0.84	2.07 ± 0.85	1.97 ± 0.75	0.363
AST	24.97 ± 11.08	25.05 ± 10.77	24.66 ± 12.24	
Total cholesterol	187.89 ± 42.24	174.13 ± 40.83	191.33 ± 41.9	0.630
Uric acid	5.82 ± 1.53	5.82 ± 1.54	5.80 ± 1.49	
Triglyceride	173.45 ± 103.29	173.38 ± 101.65	173.71 ± 109.75	
ALT	22.23 ± 11.77	22.74 ± 11.59	22.22 ± 11.80	
EGFR	73.19 ± 47.95	73.51 ± 48.13	71.95 ± 48.92	0.711
Albumin	41.42 ± 3.25	41.46 ± 3.23	41.23 ± 3.29	
High-density lipoprotein	50.58 ± 14.34	50.47 ± 14.21	51.02 ± 14.87	0.538

Abbreviations: PIR: poverty-to-income ratio; BMI: body mass index; CVD: cardiovascular disease; EGFR: estimated glomerular filtration rate; ALT: alanine aminotransferase; AST: aspartate aminotransferase.

**Table 2:** Univariate and multivariate logistic regression analyses of the training set.

	Univariate logistic regression		Multivariate logistic regression	
Characteristic	OR (95% CI)	*P* value	OR (95% CI)	*P* value
Age (years)	1.16 (1.13-1.20)	<0.001	1.16 (1.12-1.19)	<0.001
Sex, *n* (%)				
Male	Ref.		Ref.	
Female	0.60 (0.45-0.80)	<0.001	0.58 (0.41-0.80)	0.001
EGFR	1.00 (1.00-1.01)	0.031	1.00 (1.00-1.01)	0.014
Albumin	0.87 (0.84-0.91)	<0.001	0.88 (0.84-0.92)	<0.001
Race, *n* (%)				
Non-Hispanic White	Ref.		Ref.	
Non-Hispanic Black	1.00 (0.65, 1.55)	0.999	NA	NA
Other Hispanic	1.05 (0.63, 1.74)	0.851	NA	NA
Other races	1.13 (0.78, 1.63)	0.515	NA	NA
Education level, *n* (%)				
Less than high school	Ref.		Ref.	
High school diploma or GED	0.87 (0.60, 1.24)	0.865	NA	NA
More than high school	0.79 (0.54, 1.17)	0.792	NA	NA
Smoking status, *n* (%)				
Never smoker	Ref.		Ref.	
Ever smoker	1.13 (0.77, 1.66)	0.528	NA	NA
Current smoker	0.99 (0.65, 1.49)	0.952	NA	NA
Drinking status, *n* (%)				
Nondrinker	Ref.		Ref.	
Low-to-moderate drinker	1.01 (0.75, 1.36)	0.959	NA	NA
Heavy drinker	0.91 (0.55, 1.51)	0.714	NA	NA
BMI (kg/m^2^), *n* (%)				
<25.00	Ref.		Ref.	
25.00–29.99	0.70 (0.48-1.01)	0.051	0.82 (0.55-1.23)	0.344
≥30.0	0.75 (0.49, 1.16)	0.255	0.78 (0.53, 1.20)	0.297
PIR, *n* (%)				
<1.0	Ref.		Ref.	
1.0-3.0	1.30 (0.81, 2.09)	0.959	NA	NA
>3.0	1.29 (0.91, 1.85)	0.714	NA	NA
Medication use	1.07 (0.82, 1.39)	0.641	NA	NA
Hypertension	0.94 (0.69, 1.28)	0.685	NA	NA
CVD	0.80 (0.54, 1.21)	0.291	NA	NA
Antihyperlipidemic drug	1.42 (1.06-1.90)	0.019	1.18 (0.86-1.62)	0.316
Lymphocyte	1.03 (0.96, 1.10)	0.392	NA	NA
AST	1.01 (1.00-1.02)	0.030	1.01 (1.00-1.02)	0.089
Total cholesterol	1.00 (0.99, 1.00)	0.942	NA	NA
Uric acid	1.07 (0.98, 1.18)	0.147	NA	NA
Triglyceride	1.00 (0.99, 1.00)	0.813	NA	NA
ALT	0.99 (0.98, 1.01)	0.782	NA	NA
High-density lipoprotein	1.01 (0.99, 1.02)	0.389	NA	NA
Hosmer-Lemeshow test		*χ* ^2^ = 6.954	0.542	

Abbreviations: PIR: poverty-to-income ratio; BMI: body mass index; CVD: cardiovascular disease; EGFR: estimated glomerular filtration rate; ALT: alanine aminotransferase; AST: aspartate aminotransferase.

## Data Availability

All data were included in the NHANES database (https://www.cdc.gov/nchs/nhanes/index.htm).
